# Associations Between Renal Function, Uremic Toxins, and Cognitive Performance in Patients with Chronic Kidney Disease: A Pilot Study

**DOI:** 10.7150/ijms.133922

**Published:** 2026-07-30

**Authors:** Te-Wei Cheng, Kevin Li-Chun Hsieh, Chih-Chin Kao, Hsun-Hua Lee, Yen-Chung Lin, Chien-Hung Lai

**Affiliations:** 1 Department of Physical Medicine and Rehabilitation, Chang Gung Memorial Hospital, Linkou, Taoyuan 33305, Taiwan.; 2 Department of Medical Imaging, Taipei Medical University Hospital, Taipei 11031, Taiwan.; 3 Translational Imaging Research Center, Taipei Medical University Hospital, Taipei 11031, Taiwan.; 4 Department of Radiology, School of Medicine, College of Medicine, Taipei Medical University, Taipei 11031, Taiwan.; 5 Division of Nephrology, Department of Internal Medicine, Taipei Medical University Hospital, Taipei 11031, Taiwan.; 6 Division of Nephrology, Department of Internal Medicine, School of Medicine, College of Medicine, Taipei Medical University, Taipei 11031, Taiwan.; 7 Department of Neurology, Taipei Medical University Hospital, Taipei 11031, Taiwan.; 8 Department of Neurology, School of Medicine, College of Medicine, Taipei Medical University, Taipei 11031, Taiwan.; 9 Department of Physical Medicine and Rehabilitation, Taipei Medical University Hospital, Taipei 11031, Taiwan.; 10 Department of Physical Medicine and Rehabilitation, School of Medicine, College of Medicine, Taipei Medical University, Taipei 11031, Taiwan.; 11 Graduate Institute of Biomedical Optomechatronics, College of Medical engineering, Taipei Medical University, Taipei 11031, Taiwan.; 12 Taipei Medical University Research Center of Urology and Kidney (TMU-RCUK), Taipei Medical University, Taipei 11031, Taiwan.

**Keywords:** chronic kidney disease, cognitive decline, Cambridge Neuropsychological Test Automated Battery, magnetic resonance imaging

## Abstract

**Background:**

Patients with chronic kidney disease (CKD) often experience cognitive decline. However, the associations between renal dysfunction, uremic toxins, and cerebrovascular changes remain unclear.

**Aim:**

This study investigated the associations between renal function, uremic toxins, cerebrovascular imaging markers, and cognitive performance in patients with CKD plus early cognitive decline through integrated biochemical, neuropsychological, and neuroimaging assessments.

**Design:**

An observational pilot study.

**Setting:**

Outpatient clinic of the physical medicine and rehabilitation department at a tertiary hospital.

**Population:**

Adult patients with stage 3-5 CKD who exhibited early cognitive decline and could complete CANTAB-based cognitive assessments.

**Methods:**

This study included 12 patients with stage 3-5 CKD who were not undergoing hemodialysis and had early cognitive decline (Clinical Dementia Rating score: 0.5 or 1.0). Cognitive performance was assessed using the CANTAB. Serum levels of total indoxyl sulfate (IS) and total p-cresyl sulfate (pCS) were measured. Brain magnetic resonance imaging was performed to evaluate qualitative imaging markers potentially associated with cognitive impairment, such as arterial stenosis of the circle of Willis. Pearson correlations between the aforementioned biomarkers were explored.

**Results:**

Both IS and pCS levels were negatively correlated with estimated glomerular filtration rate (IS: r = -0.301, p = 0.469; pCS: r = -0.461, p = 0.250). A higher estimated glomerular filtration rate was correlated with better spatial working memory strategy performance (r = 0.695, p = 0.012). However, no clear correlation was observed between cognitive performance and arterial stenosis severity.

**Conclusion:**

This pilot study suggests that renal function is associated with selected cognitive domains in patients with CKD. The overall burden of qualitative MRI lesions was low in this cohort, although structural brain abnormalities were still observed. Integration of biochemical, neuropsychological, and brain imaging biomarkers may provide a comprehensive evaluation of cognitive function in these patients.

**Clinical Rehabilitation Impact:**

Integrating multidomain cognitive assessments with uremic toxin profiling and noninvasive brain imaging may facilitate early identification of cognitive deficits in patients with CKD, thereby supporting individualized cognitive evaluation in rehabilitation practice.

Trial Registration: NCT07272733.

## Introduction

Chronic kidney disease (CKD) is a growing global health burden, with a reported global prevalence of 9.5%. The economic burden of CKD is also substantial, with the annual cost of kidney replacement therapy for patients with end-stage renal disease (ESRD) ranging from US$20,000 to US$26,000 1. The global all-age mortality rate associated with CKD increased by 41.5% between 1990 and 2017 2. Although the all-age prevalence of CKD has increased by 29.3% since 1990, the age-standardized prevalence remains relatively stable at 1.2% 2. In Taiwan, the prevalence of CKD is approximately 11.93%. However, the public awareness of CKD remains extremely low, with only 3.54% of all affected individuals aware of their condition, particularly among those with a lower socioeconomic status 3.

The risk of cognitive decline is higher in patients with CKD or reduced renal function than in the general population 4. A meta-analysis reported that patients with CKD undergoing hemodialysis (HD) had poorer cognitive function and that patients not undergoing HD had poorer memory performance than did the general population 5. The precise mechanisms underlying cognitive decline in patients with CKD remain unclear. However, both vascular and nonvascular factors likely contribute to cognitive decline in this population 6. Vascular factors such as subclinical vascular disease, white matter lesions, and silent brain infarctions substantially contribute to cerebrovascular damage. Nonvascular contributors include depression, sleep disturbance, anemia, neurotoxins (e.g., parathyroid hormone and nitrogen metabolism byproducts), protein waste products, and increased plasma amino acid levels 7.

In patients with CKD, cognitive decline ranges in severity from mild cognitive impairment (MCI) to dementia. The risk of MCI increases with age and CKD progression. Affected cognitive domains include attention, memory, language, visuospatial capacity, executive function, and inhibitory control 8. A study reported that a lower estimated glomerular filtration rate (eGFR) was associated with poorer global cognitive function and memory performance 9. Another study used an eGFR cutoff of 60 mL/min/1.73 m^2^, which corresponds to the transition from stage 2 to stage 3 CKD, and reported that individuals with stage 3-5 CKD exhibited considerably poorer performance in orientation, attention, language, executive function, and memory than did individuals with milder CKD 10. Furthermore, a study involving children and young adults with CKD reported that participants with an eGFR of <45 mL/min/1.73 m^2^ exhibited a higher frontal white matter volume than did those with an eGFR above this threshold 11. Patients with advanced CKD (eGFR < 30 mL/min/1.73 m^2^) exhibited substantially greater declines in global cognition, naming, attention, and executive function than did patients with mild to moderate CKD 12. In general, patients undergoing HD exhibit poorer cognitive performance than does the general population, particularly in the domains of orientation, attention, and executive function. A study revealed that compared with patients with CKD not undergoing HD, those undergoing HD exhibited only modest improvements in orientation and attention 5.

Protein-bound uremic toxins (PBUTs), such as p-cresyl sulfate (pCS) and indoxyl sulfate (IS), accumulate in the circulation of patients with CKD 13. These toxins are generated through protein fermentation by the colonic microbiota 14. Because of their strong protein-binding properties, PBUTs are not completely removed during conventional HD because protein binding markedly reduces the free fraction available for dialysis. However, techniques such as hemodiafiltration and membrane adsorption may effectively remove these toxins 15. Patients with CKD exhibit both reduced cognitive function and increased serum pCS and IS levels. IS appears to be more strongly associated with cognitive deficits than pCS, although this association remains under investigation. A study associated serum IS level with executive dysfunction, particularly in patients with stage 3 CKD 16. Another study involving patients undergoing maintenance HD revealed that serum IS level was associated with poor long-term memory, mental manipulation, language ability, and spatial construction, as assessed using the Cognitive Abilities Screening Instrument 17. However, these studies relied only on neuropsychological assessments and did not incorporate brain imaging biomarkers.

Although several studies have investigated the association between impaired renal function and cognitive dysfunction, most studies have mainly used verbal cognitive assessment tools, such as the Montreal Cognitive Assessment and Mini-Mental State Examination 9,12,18. Moreover, these studies did not evaluate neuroimaging biomarkers. Neuroimaging techniques, such as cerebral blood flow assessment, may reveal the neuropathological mechanisms underlying cognitive decline in patients with ESRD 19. Jiang *et al*. noted that cerebral blood flow patterns differed between patients with ESRD and healthy individuals 20. However, few studies have investigated the associations between renal function, cognitive function, neuropsychological parameters, uremic toxins, and brain imaging biomarkers 7.

To address the aforementioned research gaps, we hypothesized that renal dysfunction, uremic toxin accumulation, and cerebrovascular imaging abnormalities would be associated with early cognitive decline in patients with CKD. Therefore, we investigated the correlations between renal function, cognitive function, uremic toxins, and cerebrovascular imaging markers in patients with CKD. First, we examined the association between renal function and PBUT levels. Next, we examined the association between renal function and cognitive performance. Finally, we examined the association between cognitive performance and brain imaging markers. Cognitive deficits in patients with CKD commonly involve orientation, attention, memory, reasoning, executive function, and global cognition 8. We used the Cambridge Neuropsychological Test Automated Battery (CANTAB) to evaluate multiple cognitive domains. Brain imaging biomarkers were evaluated using noninvasive magnetic resonance imaging (MRI). In addition, serum levels of the PBUTs IS and pCS were measured.

## Methods

### Study cohort and ethics

This study included patients with CKD who exhibited early signs of cognitive decline. The inclusion criteria were as follows: (1) being aged > 20 years, (2) undergoing regular follow-up (every 3 months) at the nephrology outpatient department of the study hospital, (3) having persistently impaired renal function (eGFR < 60 mL/min/1.73 m^2^ for ≥ 3 months, corresponding to stage 3-5 CKD), (4) having a Clinical Dementia Rating score of ≥ 0.5, and (5) being able to complete the CANTAB-based cognitive assessment. The exclusion criteria were as follows: (1) exhibiting unstable vital signs; (2) having poorly controlled hypertension, defined as resting systolic blood pressure > 180 mmHg or diastolic pressure > 110 mmHg; (3) having major cardiovascular conditions, such as aortic valve stenosis or unstable angina, or using a cardiac pacemaker; (4) having poor glycemic control; (5) having metabolic abnormalities, such as acute thyroiditis, hypokalemia, hyperkalemia, or hypovolemia; (6) having acute systemic illness or fever; or (7) having severe psychiatric disorders; (8) having more advanced cognitive impairment, defined as a Clinical Dementia Rating score > 1, or receiving antidementia medications. In addition, patients undergoing HD were excluded to minimize potential confounding effects on uremic toxin level and cognitive function. We recruited at least 20 patients between July 1, 2023, and July 1, 2024. Patients' medical records were reviewed within 1 month after enrollment to confirm the presence of preexisting conditions—specifically, hypertension, type 2 diabetes mellitus (T2DM), coronary artery disease, and dyslipidemia—before cognitive assessment.

The study procedures adhered to the ethical principles outlined in the Declaration of Helsinki. All patients provided informed consent for the publication of their clinical data. The study protocol was approved by the Institutional Review Board of Taipei Medical University (record number: TMU-JIRB-N202208048). The protocol was registered at ClinicalTrials.gov (identifier: NCT07272733). The registered trial title is “Early Detection, Remote Augmented Reality-Combined Rehabilitation Therapy and Translation Therapy for Patients with Chronic Kidney Disease and Cognitive Disorder.”

### Cognitive assessment

Cognitive function was assessed using the Cambridge Neuropsychological Test Automated Battery (CANTAB) Connect Research software (Cambridge Cognition Ltd., Cambridge, UK). CANTAB is a computerized cognitive assessment tool that evaluates several cognitive domains, including attention, memory, and executive function 21,22. Initially, all patients completed a motor screening task to familiarize themselves with the touchscreen interface. This task also served as a screening measure to determine eligibility for further assessment. Patients who passed the motor screening task subsequently completed three core tests: rapid visual (information) processing (RVP) to assess attention, delayed matching to sample (DMS) to assess memory, and spatial working memory (SWM) to assess executive function. All three tests are norm-referenced and allow standardized score analysis.

The RVP test assesses sustained attention. Participants are required to detect and respond to specific digit sequences (e.g., 2-4-6, 3-5-7, and 4-6-8) by pressing a button at the center of the screen as quickly as possible. Outcome measures include response latency, probability of false alarms, and sensitivity. The DMS test assesses simultaneous visual matching ability and short-term visual recognition memory by exploring nonverbalizable patterns. In each trial, participants are shown a complex visual pattern. After brief presentation of the pattern, participants are asked to select the exact matching pattern from four similar options either immediately (simultaneous condition) or after a delay of 0, 4, or 12 s. Outcome measures include response latency, correct response count, and error probability after a correct or incorrect response. The SWM test assesses executive function by determining whether participants can retain and manipulate visuospatial information. During the task, participants search a set of boxes to identify hidden yellow tokens, with each token located in a different box. Once a token is identified in a specific box, that box does not contain another token during the same trial. As the number of boxes increases (4, 6, 8, and 12), participants must apply a process-of-elimination strategy. Outcome measures include within-search errors (reselecting boxes already confirmed to be empty), between-search errors (revisiting boxes in which a token has already been found), and strategy score (the extent to which participants use a systematic pattern rather than a random search pattern).

### MRI

#### Image acquisition

All patients underwent non-contrast-enhanced head MRI. Images were acquired using a 3T MRI scanner (MAGNETOM Prisma; Siemens, Munich, Germany) equipped with a 20-channel head coil. The MRI protocol included T2-weighted fluid-attenuated inversion recovery imaging, three-dimensional T1-weighted magnetization-prepared rapid gradient echo-imaging, diffusion-weighted imaging, and susceptibility-weighted imaging.

#### Image analysis

The MRI data were analyzed by a board-certified neuroradiologist (KH) with 18 years of experience. The neuroradiologist was blinded to patients' clinical diagnoses, renal function, and cognitive performance. A qualitative MRI scoring system was used to assess abnormal imaging findings potentially associated with cognitive impairment. The evaluated imaging findings included (1) cerebral white matter hyperintensities, assessed using the modified Fazekas scale; (2) presence of old ischemic stroke; (3) arterial stenosis of the circle of Willis (COW); (4) presence of old petechial hemorrhage, detected using susceptibility-weighted imaging; (5) presence of cerebral amyloid angiopathy (CAA), detected using susceptibility-weighted imaging; (6) cortical atrophy; and (7) entorhinal cortical atrophy, assessed using the entorhinal cortical atrophy (ERICA) score. White matter hyperintensities were graded using the modified Fazekas scale, which classifies lesions as mild, moderate, or severe. Mild lesions (grade 1) were defined as punctate deep white matter lesions with a maximum diameter of 9 mm for single lesions and 20 mm for grouped lesions. Moderate lesions (grade 2) were defined as early confluent lesions measuring 10-20 mm for single lesions and >20 mm for grouped lesions, without connecting bridges between individual lesions. Severe lesions (grade 3) were defined as single lesions or confluent hyperintense areas measuring ≥20 mm in diameter. The presence of old ischemic stroke, old petechial hemorrhage, CAA, and cortical atrophy was scored dichotomously (Yes = 1 point; No = 0 points). Arterial stenosis of the COW was scored in accordance with vessel caliber involvement (large vessel = 2 points; small vessel = 1 point). The ERICA score ranged from 0 to 3, with 0 indicating normal volume of the entorhinal cortex and parahippocampal gyrus and 3 indicating severe atrophy in these regions. Associations between qualitative MRI findings and cognitive parameters were determined using standardized CANTAB scores 23,24.

### Laboratory tests

Laboratory tests included complete blood count, blood urea nitrogen, serum creatinine, alanine aminotransferase, electrolytes, total IS, and pCS measurements. Blood samples for IS and pCS measurements were collected in serum separator tubes. The samples were centrifuged at 3000 rpm for 10 min within 8 h after collection, and the separated serum was used for analysis. Total IS and total pCS levels were measured through liquid chromatography-tandem mass spectrometry at Chang Gung Memorial Hospital, Linkou, Taiwan. The linear analytical range for both IS and pCS was 0.05-10 μg/mL. The reference intervals were < 1.49 μg/mL for IS and < 5.85 μg/mL for pCS. The analytical accuracy was 105% for IS and 111% for pCS. The within-run and between-run imprecision values were, respectively, 1.9%-6.4% and 4.6%-7.3% for total IS and 1.1%-1.9% and 3.6%-4.5% for total pCS. All reported values represent total rather than free IS and pCS levels.

### Statistical analysis

Statistical analyses were performed using R (version 4.4.1; Posit, Boston, MA, USA). Descriptive statistics were used to summarize age, sex, comorbidity status, CKD stage, serum creatinine level, and eGFR. Pearson correlation analyses were performed to examine the correlations between individual CANTAB subtest scores, eGFR, and MRI-based assessment scores and the associations between cognitive decline, renal function, and brain abnormalities. A two-tailed p value of < 0.05 indicated statistical significance.

## Results

### Cohort characteristics

The study cohort comprised 12 patients with CKD not undergoing HD (6 men and 6 women). Their demographic characteristics are summarized in Table 1. The mean age was 75.6 ± 5.4 years, the mean serum creatinine level was 2.0 ± 1.0 mg/dL, and the mean eGFR was 34.7 ± 13.5 mL/min/1.73 m^2^. Of the patients, eight, and four had stage 3, and stage 4 CKD, respectively. The identified comorbidities included hypertension, coronary artery disease, dyslipidemia, and T2DM. Hypertension was present in all patients (12 patients), followed by T2DM (eight patients).

### Correlations of eGFR with PBUTs

**Figure 1:**
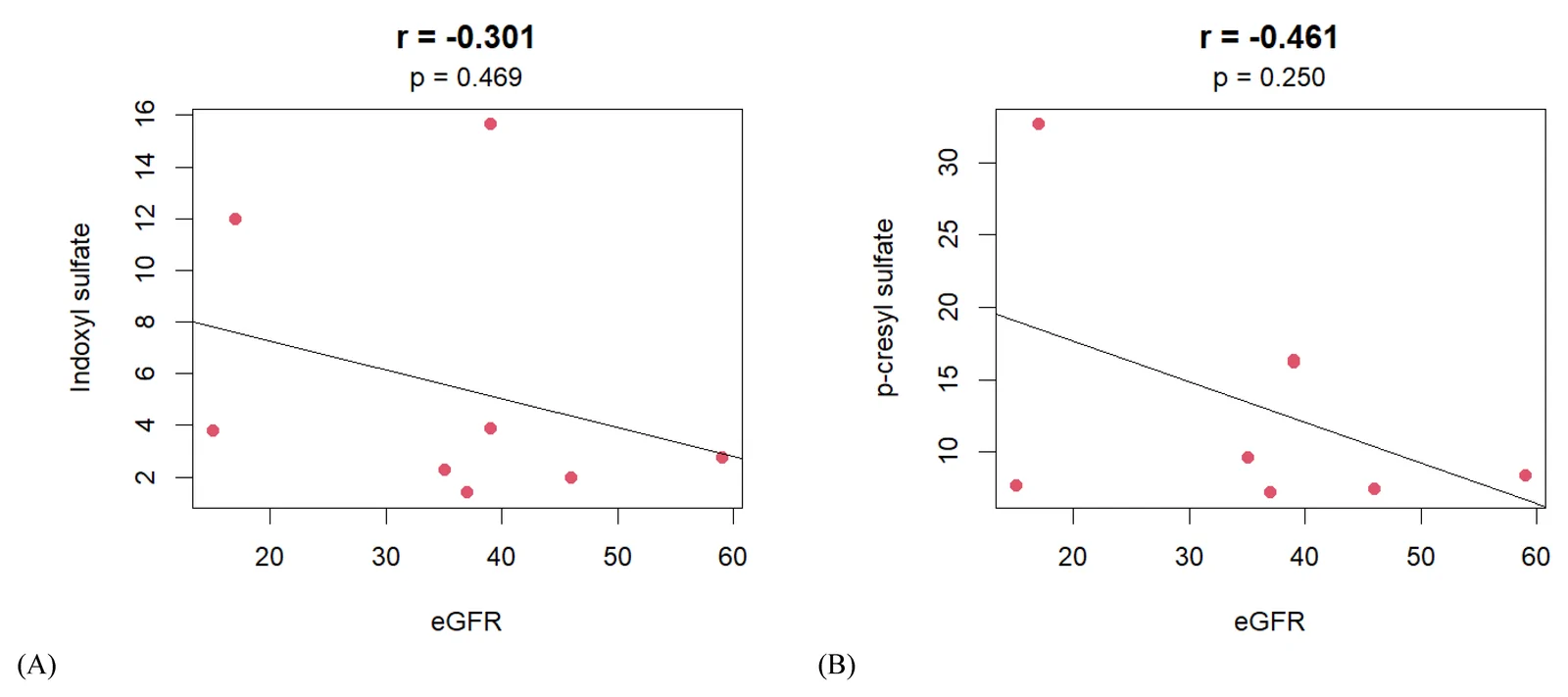
Pearson correlations between protein-bound uremic toxins and eGFR. **(A)** A negative correlation was observed between serum IS level and eGFR (r = -0.301, p = 0.469).** (B)** A negative correlation was observed between serum pCS level and eGFR (r = -0.461, p = 0.250).** Abbreviations:** eGFR, estimated glomerular filtration rate; IS, indoxyl sulfate; pCS, p-cresyl sulfate.

Serum IS and pCS levels exhibited negative but nonsignificant correlations with eGFR (IS: r = -0.301, p = 0.469; pCS: r = -0.461, p = 0.250; Figure 1A,B).

### Correlations of eGFR with various cognitive parameters

#### RVP parameters

RVP A′ indicates sensitivity to the target sequence during the RVP task. No significant correlation was observed between the raw RVP A′ score and eGFR (r = -0.210, p = 0.513). After standardization using normative data, no significant correlation was observed between the standardized RVP A′ score and eGFR (r = -0.179, p = 0.579).

**Figure 2:**
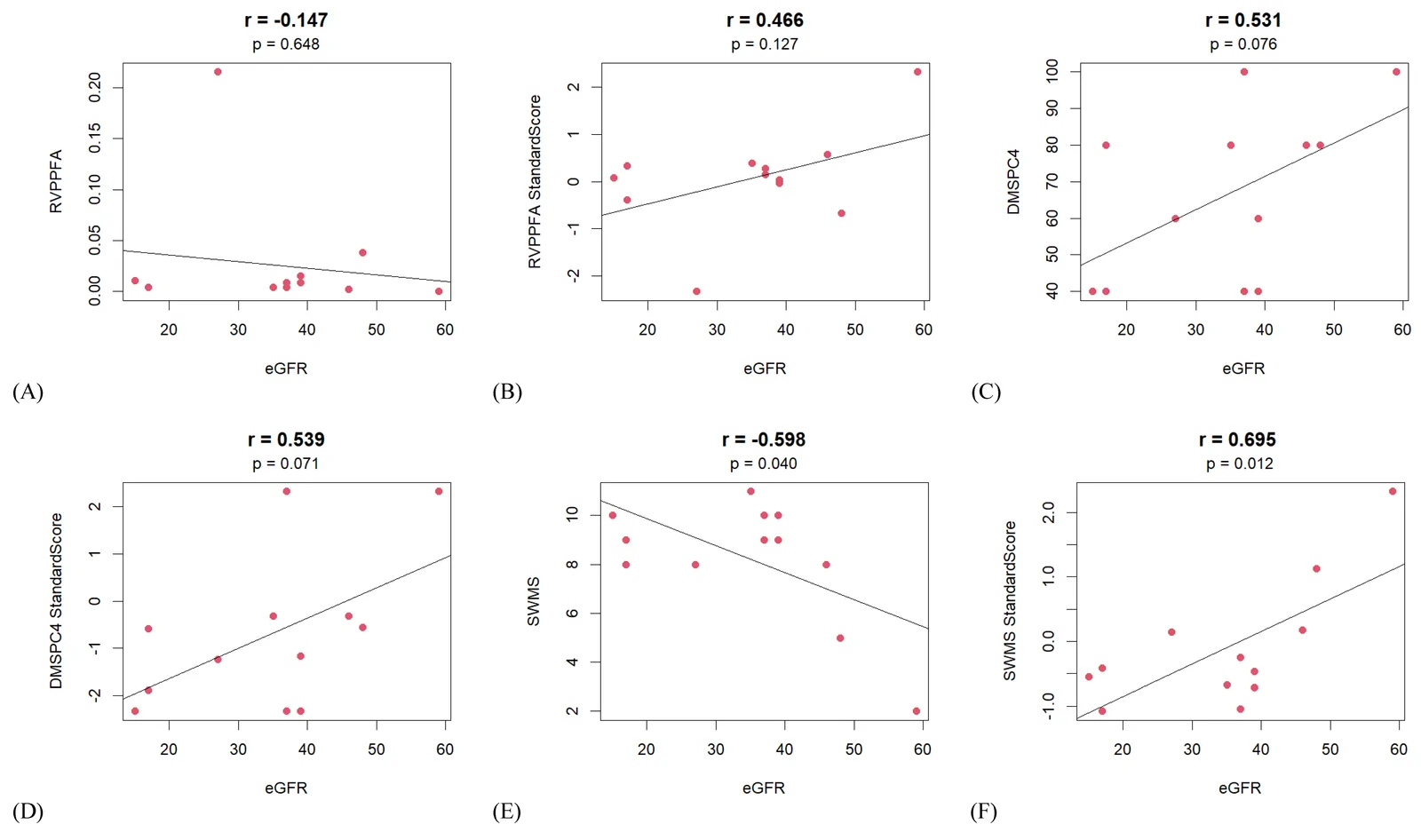
Pearson correlations between cognitive parameters and eGFR. **(A)** A negative correlation was observed between the raw RVPPFA score and eGFR (r = -0.147, p = 0.648). **(B)** A positive correlation was observed between the standardized RVPPFA score and eGFR (r = 0.466, p = 0.127). **(C)** A positive correlation was observed between the raw DMSPC4 score and eGFR (r = 0.531, p = 0.076). **(D)** A positive correlation was observed between the standardized DMSPC4 score and eGFR (r = 0.539, p = 0.071). **(E)** A significant negative correlation was observed between the raw SWMS score and eGFR (r = -0.598, p = 0.040). **(F)** A significant positive correlation was observed between the standardized SWMS score and eGFR (r = 0.695, p = 0.012). **Abbreviations:** RVPPFA, rapid visual information processing probability of false alarm; DMSPC4, delayed matching to sample percent correct 4-s delay; eGFR, estimated glomerular filtration rate; SWMS, spatial working memory strategy.

The RVP probability of false alarm (RVPPFA) is defined as the ratio of false alarms to the sum of false alarms and correct rejections. The raw RVPPFA score exhibited no significant correlation with eGFR (r = -0.147, p = 0.648; Figure 2A). After standardization using normative data, the standardized RVPPFA score also exhibited no significant correlation with eGFR (r = 0.466, p = 0.127; Figure 2B).

#### DMS parameters

DMS percent correct (DMSPC) represents the proportion of correct responses across multiple trials under each experimental condition. For trials involving simultaneous target presentation and response recording, response accuracy is expressed as DMSPC simultaneous (DMSPCS). For trials involving 0, 4, and 12 s between target presentation and response recording, response accuracy is expressed as DMSPC 0-s delay (DMSPC0), DMSPC 4-s delay (DMSPC4), and DMSPC 12-s delay (DMSPC12), respectively. Overall accuracy for all delayed conditions is expressed as DMSPC all delays (DMSPCAD). Collectively, these indices reflect visual memory performance under varying memory load conditions.

Under the simultaneous condition, no significant correlation was observed between the raw DMSPCS score and eGFR (r = -0.043, p = 0.895). After standardization using normative data, the standardized DMSPCS score exhibited no significant correlation with eGFR (r = -0.162, p = 0.615). These findings indicated that renal function was not significantly associated with performance during simultaneous matching trials.

Under the delayed conditions, the raw DMSPC0 score exhibited no significant correlation with eGFR (r = -0.249, p = 0.434). After standardization using normative data, the standardized DMSPC0 score exhibited no significant correlation with eGFR (r = -0.442, p = 0.150). The raw DMSPC4 score exhibited a positive but nonsignificant correlation with eGFR (r = 0.531, p = 0.076; Figure 2C). After standardization using normative data, the standardized DMSPC4 score showed a similar positive trend toward correlation with eGFR (r = 0.539, p = 0.071; Figure 2D). Likewise, no significant correlation was observed between eGFR and the raw DMSPC12 score (r = 0.006, p = 0.985) or standardized DMSPC12 score (r = 0.158, p = 0.625). Neither the raw DMSPCAD score nor the standardized DMSPCAD score was significantly correlated with eGFR (raw score: r = 0.203, p = 0.527; standardized score: r = 0.394, p = 0.206).

The DMS probability of error given error (DMSPEGE) indicates the probability that a participant provides an incorrect response when the preceding trial response, whether simultaneous or delayed, was also incorrect. No significant correlation was observed between eGFR and the raw DMSPEGE score (r = -0.135, p = 0.677) or standardized DMSPEGE score (r = 0.276, p = 0.385).

#### SWM parameters

The SWM strategy (SWMS) is a component of the SWM test. A lower SWMS score indicates fewer errors and corresponds to a higher standardized SWMS score. In the present study, the raw SWMS score exhibited a moderate, significant, and negative correlation with eGFR (r = -0.598, p = 0.040; Figure 2E). After standardization using normative data, the standardized SWMS score exhibited a significant and positive correlation with eGFR (r = 0.695, p = 0.012; Figure 2F). These findings indicated that a higher eGFR was associated with a lower raw SWMS score and a higher standardized SWMS score.

No significant correlation was observed between eGFR and SWM between-search errors in trials involving four, six, or eight tokens (r = 0.029, p = 0.928). Even after standardization using normative data, the correlation remained nonsignificant (r = 0.028, p = 0.932). These findings suggest that renal function was not significantly associated with revisiting errors during the SWM task.

### MRI findings

**Figure 3:**
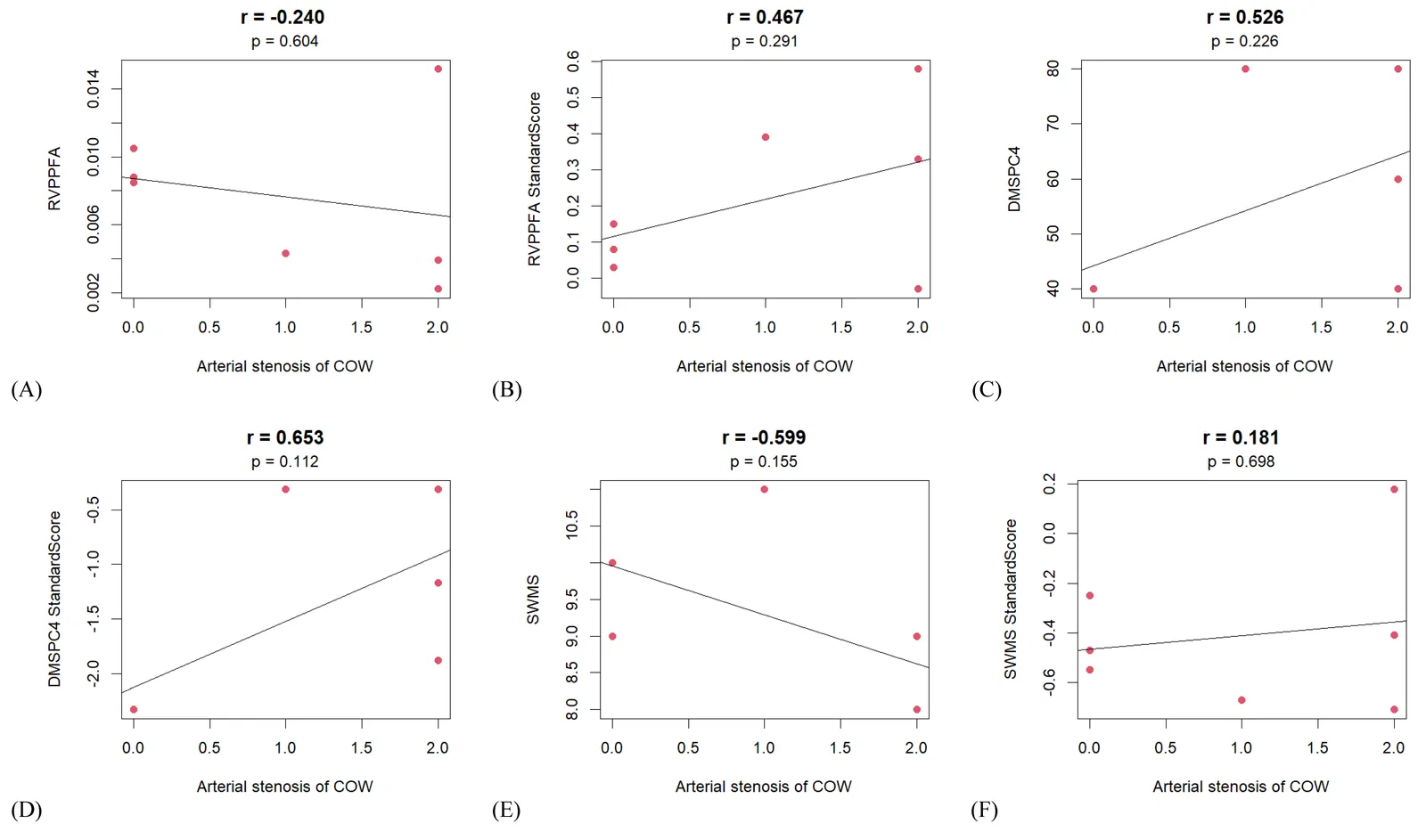
Pearson correlations between cognitive parameters and arterial stenosis of the circle of Willis. **(A)** No significant correlation was observed between the raw RVPPFA score and arterial stenosis severity (r = -0.240, p = 0.604). **(B)** No significant correlation was observed between the standardized RVPPFA score and arterial stenosis severity (r = 0.467, p = 0.291). **(C)** No significant correlation was observed between the raw DMSPC4 score and arterial stenosis severity (r = 0.526, p = 0.226). **(D)** No significant correlation was observed between the standardized DMSPC4 score and arterial stenosis severity (r = 0.653, p = 0.112). **(E)** No significant correlation was observed between the raw SWMS score and arterial stenosis severity (r = -0.599, p = 0.155). **(F)** No significant correlation was observed between the standardized SWMS score and arterial stenosis severity (r = 0.181, p = 0.698).** Abbreviations:** RVPPFA, rapid visual information processing probability of false alarm; DMSPC4, delayed matching to sample percent correct 4-s delay; SWMS, spatial working memory strategy.

The median MRI scores were as follows: modified Fazekas scale, 1 (interquartile range [IQR]: 1-2); old ischemic stroke, 0 (IQR: 0-1); arterial stenosis of the COW, 1 (IQR: 0-2); old petechial hemorrhage, 1 (IQR: 0-1); CAA, 0 (IQR: 0-0); cortical atrophy, 1 (IQR: 1-1); and ERICA score, 1 (IQR: 0-1). Associations between qualitative MRI findings and selected standardized CANTAB cognitive parameters, such as RVPPFA, DMSPC4, and SWMS, were explored. Overall, the burden of MRI lesions was low in our cohort. Because arterial stenosis of the COW may lead to cerebrovascular insufficiency, correlations between arterial stenosis severity and cognitive performance were investigated (Figure 3). No significant association was observed between arterial stenosis severity and the raw RVPPFA score (r = -0.240, p = 0.604; Figure 3A) or standardized RVPPFA score (r = 0.467, p = 0.291; Figure 3B). Similarly, arterial stenosis severity was not significantly associated with the raw DMSPC4 score (r = 0.526, p = 0.226; Figure 3C), standardized DMSPC4 score (r = 0.653, p = 0.112; Figure 3D), raw SWMS score (r = -0.599, p = 0.155; Figure 3E), or standardized SWMS score (r = 0.181, p = 0.698; Figure 3F). Overall, no significant or consistent associations were observed between arterial stenosis of the COW and the examined cognitive parameters.

### Results of subgroup analyses

Subgroup correlation analyses by hypertension, T2DM, and CKD stage were performed to explore potential confounding effects (Supplementary Table 1). Because all patients in the cohort had hypertension, the hypertension subgroup analysis was equivalent to the overall cohort analysis. In addition, age-stratified comparisons were performed by dividing participants into two groups by using 75 years as the cutoff. No significant differences in CANTAB cognitive indices were observed between participants aged > 75 years and those aged ≤ 75 years. In the subgroup analyses, significant associations were observed between eGFR and the standardized DMSPEGE score in patients with T2DM (n = 8; r = 0.740, p = 0.036). Among patients with stage 3 CKD (n = 8), significant associations were observed between eGFR and several standardized CANTAB outcomes, including RVPA (r = 0.854, p = 0.007), DMSPCAD (r = 0.767, p = 0.026), DMSPCS (r = 0.819, p = 0.013), DMSPC12 (r = 0.766, p = 0.027), DMSPEGE (r = 0.839, p = 0.009), and SWMS (r = 0.959, p < 0.001) scores). However, because of the limited sample size in each subgroup and the exploratory nature of these analyses, the findings should be interpreted with caution.

## Discussion

We investigated the associations between cognitive performance, renal function, and brain imaging biomarkers. Cognitive performance was evaluated using three CANTAB tests: DMS, SWM, and RVP. Arterial stenosis and collateral circulation of the COW were assessed using noninvasive MRI.

The PBUTs pCS and IS are low-molecular-weight compounds. pCS is a phenol-derived compound with a molecular weight of 188 Da, and IS is an indole derivative with a molecular weight of 212 Da. These solutes are predominantly eliminated through tubular secretion during urine formation, which is a key pathway for PBUT clearance. However, IS and pCS tend to accumulate in patients with CKD. Conventional HD is ineffective in removing these toxins because of their high protein-binding affinity; therefore, hemofiltration is required for effective clearance 25-27. In the present study, impaired renal function was associated with elevated circulating levels of these PBUTs. This finding is consistent with the known accumulation of these solutes in patients with reduced eGFR. It is also consistent with the findings of Suchy-Dicey *et al*. 28, who reported that the clearance of both IS and pCS was significantly correlated with eGFR. Notably, reduced pCS clearance is associated with an increased risk of mortality in patients with CKD, independent of eGFR 28. eGFR reflects overall renal function and primarily represents glomerular filtration capacity. However, eGFR does not specifically measure tubular secretory function. Nevertheless, it may partially reflect tubular secretory capacity.

In addition to vascular mechanisms, the neurodegenerative hypothesis of the kidney-brain axis provides an additional framework for understanding cognitive dysfunction in CKD. Uremic toxicity, oxidative stress, and inflammation have been proposed as key contributors to neuronal injury in patients with CKD 29. PBUTs, particularly IS and pCS, have been associated with oxidative stress, neuroinflammation, and neuronal dysfunction 30,31. Exploratory biomarker studies involving patients with CKD and cognitive dysfunction have revealed Alzheimer's disease (AD)-like peripheral molecular alterations, such as abnormal amyloid precursor protein processing, altered amyloid-β expression, tau-related changes, inflammatory cytokine changes, and cholinergic biomarker abnormalities 32,33. These findings indicate that CKD-associated cognitive impairment involves oxidative and inflammatory stress-related nonvascular neurodegenerative pathways in addition to cerebrovascular insufficiency.

A study reported that DMSPCAD scores were lower in patients with amnestic MCI than in healthy controls and individuals with subjective cognitive decline 34. Another study reported that higher amyloid-β levels in the cerebrospinal fluid were associated with lower DMSPCAD scores in patients with MCI 35. Blackwell *et al*. 36 noted substantial differences in CANTAB DMS test performance between individuals who subsequently developed AD and those who did not. This finding highlights the potential utility of the DMS test as an early neuropsychological marker of AD-related cognitive decline. Another study reported substantial differences in DMS test performance between individuals with probable AD and those without dementia, between patients with MCI and healthy controls, and between patients with AD and healthy individuals 37. In the present study, neither the raw nor standardized DMSPCAD score was significantly correlated with eGFR. However, the raw DMSPC4 score showed a positive but nonsignificant correlation with eGFR, and the standardized DMSPC4 score showed a similar positive trend. These findings suggest that the association between renal function and short-term visual recognition memory was modest and did not reach statistical significance. A prospective cohort study demonstrated strong associations between eGFR and changes in visual memory, assessed using the learning slope of the Benton Visual Retention Test, and verbal learning, assessed using the California Verbal Learning Test. A higher eGFR was associated with a slower cognitive decline 38. Visual working memory refers to the ability to actively maintain visual information while processing information essential for completing ongoing tasks 39,40. Consistent with this concept, early impairment of working memory has been reported in other neurodegenerative disorders. For example, patients with early-stage Parkinson's disease exhibit altered working memory-related neurocognitive performance even in the absence of overt dementia, which suggests that working memory is particularly vulnerable during the early stages of neurodegenerative disease 41. A study reported that visual memory performance, assessed using the Benton Visual Retention Test, was inversely correlated with amyloid-β burden in brain regions commonly affected in AD 42. Because amyloid-β accumulation in AD preferentially impairs visual short-term memory, the association observed in the present study between a higher eGFR and better cognitive performance under delayed DMS test conditions suggests that preserved renal function is associated with the preservation of memory processes vulnerable to AD-related pathological changes.

SWM refers to the capacity to actively maintain and manipulate spatial information in working memory over short periods. The right posterior parietal cortex and right dorsolateral prefrontal cortex are key brain regions associated with SWM 43. In patients with ESRD undergoing HD, blood oxygen level-dependent functional MRI revealed altered activation patterns in the frontal cortex and parietal lobule, which are key brain regions associated with SWM. Lower activity levels in these regions were associated with poorer cognitive performance 44. In the present study, a higher eGFR was significantly associated with better performance on the SWM test, suggesting that preserved renal function is associated with efficient executive and visuospatial processing. Notably, we conducted only one SWM test per patient, which minimized potential confounding by practice effects. Cacciamani *et al*. 45 reported that repeated SWM testing at 6- and 12-month follow-up assessments in patients with MCI resulted in major practice-related improvements in cognitive performance. Therefore, the SWM differences observed in the present study are less likely to be attributable to practice effects.

To address potential confounding effects, we performed exploratory analyses for age, hypertension, T2DM, and CKD stage. Age-stratified comparisons performed using 75 years as the cutoff revealed no significant differences in CANTAB cognitive indices. We further performed subgroup correlation analyses by hypertension status, T2DM status, and CKD stage. Because hypertension was present in all patients, analyses of the hypertension subgroup were equivalent to those of the overall cohort. Because the numbers of patients without T2DM were considerably small, interpretation focused on clinically relevant subgroups with sufficient case counts. Notably, the association between eGFR and SWMS Standard Score was observed in the overall cohort and was also evident in the CKD stage 3 subgroup, suggesting a potentially consistent relationship between renal function and spatial working memory in this cohort. In addition, the CKD stage 3 subgroup showed significant associations between eGFR and several standardized CANTAB outcomes, including RVPA, DMSPCAD, DMSPCS, DMSPC12, DMSPEGE, and SWMS scores. These findings may suggest that kidney-brain associations are detectable even in earlier stages of CKD, when subtle differences in renal function may still be related to cognitive performance. However, the associations were not uniform across all cognitive indices, indicating that different neuropsychological domains or CANTAB measures may show different sensitivities to renal function. Nevertheless, because of the small size of the analytical sample and the exploratory nature of the analyses, the findings should be interpreted with caution and larger sample size studies are required to verify these results.

Brain atrophy, white matter lesions, and vascular insufficiency contribute to cognitive decline and dementia in patients with CKD 46. These contributions may be partly explained by the shared perfusion characteristics and physiological features of the kidneys and brain, often referred to as the kidney-brain axis. Both organs operate under relatively high perfusion pressure and depend on steep pressure gradients to maintain stable blood flow, which increases their vulnerability to hypertensive injury 29. Experimental and clinical data suggest that the kidneys and brain participate in bidirectional molecular cross-talk mediated by inflammatory mediators, oxidative stress, and neurohumoral pathways, which may exacerbate vascular and neuronal injury 47. In the present study, no consistent association was observed between arterial stenosis of the COW and cognitive performance. This finding may be partly explained by the early cognitive status of our cohort and the relatively low burden of structural brain abnormalities on MRI. Patients with internal carotid artery stenosis have been reported to exhibit cognitive deficits, particularly in domains related to frontal executive function 48. Chronic hypoperfusion caused by high-grade internal carotid artery stenosis has been proposed as a key mechanism underlying such deficits. In patients with asymptomatic unilateral carotid stenosis, ipsilateral cerebral hypoperfusion has been demonstrated using perfusion-weighted MRI and duplex ultrasonography 49. High-grade asymptomatic carotid stenosis accompanied by inadequate collateral circulation in the COW has been associated with poor cognitive performance, particularly in learning and recall, attention, and working memory 50. Nevertheless, mild structural brain abnormalities were frequently observed in our cohort of patients with early cognitive decline (Clinical Dementia Rating score: 0.5 or 1.0). Qualitative MRI revealed mild to moderate white matter hyperintensities, reflected by a modified Fazekas score of 1 or 2, and cortical atrophy was common. Therefore, MRI-detectable brain abnormalities may already be present in patients with CKD during the early stages of cognitive decline. Nonetheless, larger cohorts and more quantitative imaging approaches are required to clarify the associations between these abnormalities and specific cognitive domains.

This study has some limitations. First, the sample size was relatively small, which may limit the generalizability of our findings and increase the risk of type II error. Thus, larger studies are required to validate the results. Second, because of the limited sample size, we did not perform multivariate analyses to adjust for potential confounders such as age, hypertension, T2DM, and CKD stage. Accordingly, the findings should be interpreted with caution. Third, we did not investigate the effects of rehabilitative or cognitive training interventions on CKD-related cognitive decline. Nevertheless, this pilot study identified associations between renal function and specific cognitive domains, particularly SWM. These findings may inform future targeted interventions. Finally, although no consistent associations were observed between arterial stenosis of the COW and CANTAB performance in our patients, structural brain abnormalities were still noted despite the overall low burden of qualitative MRI lesions. Furthermore, the MRI-based evaluation relied on a qualitative scoring system, which may not fully capture quantitative or perfusion-related abnormalities. Future studies should include larger cohorts spanning a broader range of cognitive impairment severity, from early cognitive decline to more advanced impairment, and should incorporate quantitative perfusion measures to further clarify the correlations between MRI-detectable brain abnormalities, renal dysfunction, and specific cognitive domains in patients with CKD.

## Conclusion

To the best of our knowledge, the present study is the first to integrate renal function evaluation, uremic toxin measurement, neuropsychological assessment, and brain MRI to characterize early cognitive decline in patients with CKD. Renal function was associated with the selected cognitive performance measures in patients with CKD. Specifically, better renal function was associated with better performance in SWM. These preliminary findings suggest a kidney-brain relationship in patients with CKD. However, arterial stenosis of the COW was not consistently associated with cognitive performance. Although mild MRI-detectable brain abnormalities were observed, indicating that structural brain changes may occur during the early stages of cognitive decline in patients with CKD, the overall burden of qualitative MRI lesions was low in this cohort. Integration of biochemical, neuropsychological, and brain imaging biomarkers may facilitate early detection of and comprehensive assessment of cognitive decline in these patients. Given the pilot nature of this study, additional investigations involving larger sample sizes and a broader spectrum of CKD severity are required to provide more robust evidence.

## Supplementary Material

Supplementary table.

## Figures and Tables

**Table 1:** Patient demographics

Case number (trial ID)	Age (years)	Sex	Comorbidity	Serum creatinine level (mg/dL), mean ± SD or n	eGFR (mL/min/1.73 m^2^), mean ± SD or n	CKD stage*
Overall	75.6 ± 5.4	F/M, n = 6/6	-	2.0 ± 1.0	34.7 ± 13.5	G3/G4: 8/4
1 (001)	82	F	HTN, CAD, and DLD	1.5	35	3
2 (003)	77	F	HTN and DLD	1.2	46	3
3 (004)	71	F	HTN, CAD, DLD, and T2DM	3.2	15	4
4 (005)	79	M	HTN, CAD, DLD, and T2DM	3.7	17	4
5 (008)	82	M	HTN, DLD, and T2DM	1.8	39	3
6 (009)	69	F	HTN	1.5	37	3
7 (011)	77	M	HTN, CAD, DLD, and T2DM	1.8	39	3
8 (013)	75	F	HTN and T2DM	1.9	27	4
9 (015)	80	M	HTN and CAD	3.7	17	4
10 (017)	75	M	HTN, CAD, and T2DM	1.9	37	3
11 (018)	76	M	HTN, CAD, DLD, and T2DM	1.5	48	3
12 (020)	64	F	HTN, DLD, and T2DM	1	59	3

**Abbreviations:** CAD, coronary artery disease; CKD, chronic kidney disease; DLD, dyslipidemia; eGFR, estimated glomerular filtration rate; F, female; HTN, hypertension; M, male; n, number; SD, standard deviation; T2DM, type 2 diabetes mellitus. *CKD stage was determined on the basis of a documented clinical diagnosis of persistent renal function impairment rather than a single eGFR measurement at enrollment and was classified as per the KDIGO 2024 Clinical Practice Guideline (G3: 30-59 mL/min/1.73 m^2^, G4: 15-29 mL/min/1.73 m^2^, and G5: <15 mL/min/1.73 m^2^).

## Data Availability

The datasets generated during the present study are available from the corresponding author upon reasonable request.
