# Supplementary material for: Associations Between Renal Function, Uremic Toxins, and Cognitive Performance in Patients with Chronic Kidney Disease: A Pilot Study
Source: Int J Med Sci. 2026 Jul 30;23(9):2899–910. doi: 10.7150/ijms.133922 (PMC13539910; doi:10.7150/ijms.133922)
Supplement: Supplementary file 1 [file ijmsv23p2899s1.pdf]

Supplementary Table 1. Subgroup analysis

	**n = 12**	**HTN (n=12)**	**DM2 (n = 8)**	**CKD stage 3 (n = 8)**
eGFR versus pCS	r = −0.461, p = 0.250	r = −0.461, p = 0.250	r = −0.426, p = 0.475	r = −0.426, p = 0.475
eGFR versus IS	r = −0.301, p = 0.469	r = −0.301, p = 0.469	r = −0.240, p = 0.698	r = −0.240, p = 0.698
eGFR versus standardized RVPA score	r = −0.179, p = 0.579	r = −0.179, p = 0.579	r = −0.044, p = 0.918	r = 0.854, **p = 0.007**
eGFR versus standardized RVPPFA score	r = 0.466, p = 0.127	r = 0.466, p = 0.127	r = 0.438, p = 0.278	r = 0.641, p = 0.087
eGFR versus standardized DMSPCAD score	r = 0.394, p = 0.206	r = 0.394, p = 0.206	r = 0.533, p = 0.173	r = 0.767, **p = 0.026**
eGFR versus standardized DMSPCS score	r = −0.162, p = 0.615	r = −0.162, p = 0.615	r = 0.094, p = 0.825	r = 0.819, **p = 0.013**
eGFR versus standardized DMSPC0 score	r = −0.442, p = 0.150	r = −0.442, p = 0.150	r = −0.287, p = 0.491	r = 0.120, p = 0.777
eGFR versus standardized DMSPC4 score	r = 0.539, p = 0.071	r = 0.539, p = 0.071	r = 0.651, p = 0.080	r = 0.479, p = 0.230
eGFR versus standardized DMSPC12 score	r = 0.158, p = 0.625	r = 0.158, p = 0.625	r = 0.192, p = 0.649	r = 0.766, **p = 0.027**
eGFR versus standardized DMSPEGE score	r = 0.276, p = 0.385	r = 0.276, p = 0.385	r = 0.740, **p = 0.036**	r = 0.839, **p = 0.009**
eGFR versus standardized SWMBE468 score	r = 0.028, p = 0.932	r = 0.028, p = 0.932	r = 0.240, p = 0.567	r = 0.483, p = 0.226
eGFR versus standardized SWMS score	r = 0.695, **p = 0.012**	r = 0.695, **p = 0.012**	r = 0.682, p = 0.063	r = 0.854, **p = 0.007**

Abbreviations: eGFR, estimated glomerular filtration rate; T2DM, type 2 diabetes mellitus; HTN, hypertension; CKD, chronic kidney disease; pCS, p−cresyl sulfate; IS, indoxyl sulfate; RVPA, rapid visual information processing A′; RVPPFA, rapid visual information processing probability of false alarm; DMSPCAD, delayed matching to sample percent correct all delays; DMSPCS, delayed matching to sample percent correct simultaneous; DMSPC0, delayed matching to sample percent correct 0−s delay; DMSPC4, delayed matching to sample percent correct 4−s delay; DMSPC12, delayed matching to sample percent correct 12−s delay; DMSPEGE, delayed matching to sample probability of error given error; SWMBE468, spatial working memory between errors (4−, 6−, and 8−token stages); SWMS, spatial working memory strategy.

Because of the small sample size, the subgroup analyses were exploratory in nature, and their results should be interpreted with caution.
